# A systematic review of flurbiprofen 8.75 mg dose and risk of adverse events (excluding haemorrhagic) resulting from drug-drug interactions

**DOI:** 10.3389/fphar.2024.1107185

**Published:** 2024-03-06

**Authors:** Alison Evans, Debabrata Roy, Sandeep Dhanda, Samantha Lane, Graça Coutinho, Anuradha Kulasekaran, David Miller-Shakesby, Nagalakshmi Ramamoorthi, Saad Shakir

**Affiliations:** ^1^ Drug Safety Research Unit, Southampton, United Kingdom; ^2^ University of Portsmouth, Portsmouth, United Kingdom; ^3^ Global Medical Affairs, Reckitt Benckiser Health Limited, Slough, United Kingdom; ^4^ Risk Management and Vigilance, Reckitt Benckiser Health Limited, Slough, United Kingdom

**Keywords:** flurbiprofen, lozenge, oromucosal spray, drug-drug interactions, adverse events

## Abstract

**Background:** Flurbiprofen 8.75 mg lozenges and oromucosal sprays are used for symptomatic relief of sore throat in patients aged 12 years and over. The documented adverse events of flurbiprofen use include those related to its pharmacological actions, namely, increased risk of haemorrhagic events, however other adverse events (such as nephrotoxicity and cardiac failure) have been known to occur. The likelihood of occurrence of adverse events increases when flurbiprofen is used concomitantly with some other medications. Therefore, the objective of this systematic review was to collate the current evidence on adverse events which occur with flurbiprofen 8.75 mg dose (any formulation), in particular as a result of interaction with other medicinal products, with a focus on non-haemorrhagic events.

**Methods:** Systematic searches of the literature were conducted to identify literature on any formulation of flurbiprofen 8.75 mg up to the date of the electronic database search (data lock: 28 April 2020). Publications were screened to identify studies reporting non-haemorrhagic adverse events with flurbiprofen 8.75 mg and/or non-haemorrhagic adverse events in the comparator arm. Data extraction was performed for eligible studies according to pre-defined criteria and summarised in narratives, tables and figures. Risk of bias and certainty of evidence assessments were planned for each included study where results relating to the primary objective of the systematic review were available.

**Results:** Of 1,528 publications identified by systematic literature searches, 26 met the inclusion criteria and were included in this review. None of these 26 studies contained information on non-haemorrhagic adverse events occurring as a result of a drug-drug interaction (interaction with concomitant medication used with flurbiprofen 8.75 mg), as per the primary objective and secondary objectives of the systematic review.

**Conclusion:** Results from this systematic review on the risk of non-haemorrhagic events did not provide evidence for these events occurring as a result of interaction with other medicinal products. Additional appropriately designed studies would be required to confirm whether these findings suggest a true absence of risk or limitations in reporting.

## Introduction

Flurbiprofen is a non-steroidal anti-inflammatory drug (NSAID) which is used widely for its analgesic effect. Flurbiprofen is available as a tablet for treatment of musculoskeletal pain (including arthritis and osteoarthritis) and for the relief of mild to moderate pain including dental and post-operative pain (Reckitt Benckiser Healthcare UK Ltd, 2016). When applied to the pharynx, the local and systemic effects of flurbiprofen provide symptomatic relief of sore throat; this led to flurbiprofen 8.75 mg being manufactured in different formats, such as lozenge and spray (Reckitt Benckiser Healthcare UK Ltd, 2016). A single dose of flurbiprofen 8.75 mg (lozenge or oromucosal spray) has been demonstrated to provide relief for sore throat, through a significant reduction in pain intensity, difficulty swallowing, and sensations associated with swelling of the throat (Reckitt Benckiser Healthcare UK Ltd, 2016; Reckitt Benckiser Healthcare UK Ltd, 2018).

Flurbiprofen lozenge was first licensed in the European Union (EU) in 2006 for the short-term symptomatic relief of sore throat in adults and children over the age of 12 years. The oromucosal spray is licensed for use in adults aged 18 years and over (Reckitt Benckiser Healthcare UK Ltd, 2016; Reckitt Benckiser Healthcare UK Ltd, 2018).

A well-documented adverse event related to NSAID use is haemorrhage. NSAIDs, including flurbiprofen, inhibit the cyclooxygenase (COX) enzyme, specifically the COX-1 enzyme which is involved in gastroprotection and platelet aggregation for blood clot formation (Rao and Knaus, 2008; Moore et al., 2015). Consequently, the inhibition of COX-1 can result in an increase in the risk of bleeding, primarily gastrointestinal (GI) bleeding.

In addition to rare haemorrhagic events, a range of other adverse events (excluding haemorrhagic) are listed in the summary of product characteristics (SmPC); in many instances, these adverse events are a result of drug-drug interactions (DDIs) between flurbiprofen and other medicinal products. DDIs are known to occur between flurbiprofen and other NSAIDs, antihypertensive drugs, and cardiac glycosides which can result in adverse events such as nephrotoxicity, GI ulceration, and cardiac events (Reckitt Benckiser Healthcare UK Ltd, 2016). Furthermore, flurbiprofen may alter serum levels of concomitant medications resulting in the potential risk of increased toxicity or altered effectiveness of the medication (Reckitt Benckiser Healthcare UK Ltd, 2016). Examples of such medications are listed in the SmPC and include lithium and phenytoin (Reckitt Benckiser Healthcare UK Ltd, 2016). Thus, it is important to consider usage of concomitant medication in the evaluation of any potential risk of adverse events occurring with flurbiprofen (Reckitt Benckiser Healthcare UK Ltd, 2016).

The objective of the systematic review was to collate the current evidence on adverse events which occur with flurbiprofen 8.75 mg dose (any formulation), in particular as a result of interaction with other medicinal products. The systematic review was conducted in two parts according to event type: haemorrhagic and non-haemorrhagic adverse events. The results focusing on haemorrhagic events have been previously published (Dhanda et al., 2021). This paper focuses on non-haemorrhagic adverse events that occur in patients taking flurbiprofen 8.75 mg dose (any formulation).

The primary objective of this part of the systematic review was to identify the frequency of all non-haemorrhagic adverse events occurring as a result of concomitant use of flurbiprofen 8.75 mg dose (any formulation) with other medicinal products (i.e., DDIs). Secondary objectives included describing the nature of the drug interactions (i.e., class of drug interacting with flurbiprofen 8.75 mg), the severity of all non-haemorrhagic adverse events, and comparing the occurrence of non-haemorrhagic adverse events with flurbiprofen 8.75 mg dose (any formulation) in combination with other medicinal products to comparator arms (e.g., other NSAIDs) of studies where a comparator group is available.

## Methods

### Inclusion criteria for studies

The following relevant study designs were included: clinical trials (randomised and non-randomised, blinded and non-blinded), cohort studies (prospective and retrospective), case-control studies, cross sectional studies, case series and case reports.

This review was conducted according to the Preferred Reporting Items for Systematic Reviews and Meta-Analyses (PRISMA) statement and the inclusion criteria were in line with the PICOS (Participants, Interventions, Comparators, Outcomes, and Study design) categories (Supplementary Material: Inclusion criteria) (Liberati et al., 2009; Page et al., 2021).

### Exclusion criteria for studies

Studies in languages other than English, studies only specifying haemorrhagic events, pre-clinical studies, reviews (although references from any appropriate reviews were checked for any other papers that were eligible for inclusion), conference abstracts.

### Information sources and search strategy

Systematic searches of the literature were conducted using methods previously described (Dhanda et al., 2021). Briefly, PubMed/MEDLINE, Embase, The Cochrane Library, Web of Science, ClinicalTrials.gov (http://clinicaltrials.gov), and EU Clinical Trials Register (https://www.clinicaltrialsregister.eu/ctr) were searched up to 28 April 2020 to identify literature on flurbiprofen 8.75 mg (any formulation). No restriction on dates or coverage were applied.

The following search strategy was used where possible:

Search concept 1:

‘Flurbiprofen (including all synonyms for this concept)’

AND.

Search concept 2:

‘Lozenge OR oromucosal spray (including all synonyms for these concepts, e.g., spray, buccal, oromucosal) OR 8.75’.

A manual search of reference lists from included literature was conducted to identify additional research not picked up in the initial search. Further searches were conducted for case reports in the publicly available EudraVigilance database and the European Medicines Agency (EMA) website.

Full details of the search strategy is included in the Supplementary Material: Search Strategy.

In addition, in order to include the most recent data for this publication, a literature review was conducted using the same search criteria to cover the period between 29 April 2020 to 14 February 2023.

### Study selection

Following de-duplication of studies identified during the electronic search, the resulting studies were reviewed independently by two reviewers. Any discrepancies were adjudicated by a third reviewer where necessary. Reasons for exclusion were recorded. References were managed in EndNote (X7.8) (apart from studies identified via ClinicalTrials.gov and EU Clinical Trials, for which export to EndNote was not possible).

### Data extraction

For eligible studies, data was extracted independently by the two reviewers into a data extraction form (Table 1). Where data was not available, this was recorded as ‘not specified’.

**TABLE 1:** Data items for extraction.

Data	Result
Information on paper	
Study ID	Allocated by reviewer
Name of first author	
Year of publication	
Title of paper	
Link to local pdf copy or hyperlink to online copy	
Study design	
Type of study (e.g., RCT, cohort, case control)	Primary, secondary care
Country	
Setting of study	
Follow-up duration (if applicable)	
Population studied	
Specific age criteria to study population (if any)	
Medical indication for treatment	
Exposure of interest	
Flurbiprofen (dose confirmation, formulation)	
Comparator	
Placebo or active comparator	
If active - class of medicinal product, name, dose, formulation, indication for prescribing, category of sales (GSL, PO, POM), alone or in combination with other medicinal product	
Baseline results	
Total sample size	
Number of participants taking flurbiprofen	
Number of participants taking comparator	
Age of participants	
Gender	
Relevant co-morbidities	
Method of statistical analysis	
Crude/adjusted measures	
Modelling	
Adjustment for confounding	
Outcomes^a^	
Frequency of events and in which group of patients (exposure or comparator if comparator applicable)	
Details of events—site, severity, clinical sequelae	
Details of whether event occurred with exposure or comparator alone or whether in combination with other medicinal products (i.e., DDI’s)	
If occurred in combination with other medicinal product - details of concomitant medications (class, dose, formulation, indication)	
Measure of frequency/effect	
Risk of event (in either group)	
Rate/odds of event (if provided)	
Measure of effect (crude and/or adjusted)—risk ratio, rate ratio, odds ratio, hazard ratio (depending on type of study and what has been reported)	
Confidence interval (if provided) *p*-value (if provided)	
Author reported causality^b^	

^a^ Outcomes reported as specified by authors in the individual studies.

^b^ Author reported causality will be extracted if provided; otherwise individual causality assessments will not be performed as part of this systematic review.

### Risk of bias assessment and certainty of evidence

It was planned that each included study would be assessed for risk of bias and certainty of evidence where results relating to the primary objective of the systematic review were available. The aim was to use the Cochrane Risk-of-Bias (RoB2) tool and the Risk Of Bias In Non-randomised Studies–of Interventions (ROBINS-I) tool to assess risk of bias in the results of randomised trials and non-randomised studies, respectively (Sterne et al., 2016; Sterne et al., 2019), according to the guidelines outlined in the Cochrane Handbook (Higgins and Thomas, 2019), and Grading of Recommendations, Assessment, Development and Evaluations (GRADE) framework to conduct certainty of evidence assessments (Balshem et al., 2011).

### Synthesis of results

Results are summarised in narratives, tables and figures. Treatments and outcomes reported in individual studies are presented as specified by the authors. An attempt was made to calculate measures of frequency (e.g., risk) in studies where only counts were specified (where the appropriate numerator and denominator information was provided).

## Results

### Results of systematic literature searches

**FIGURE 1:**
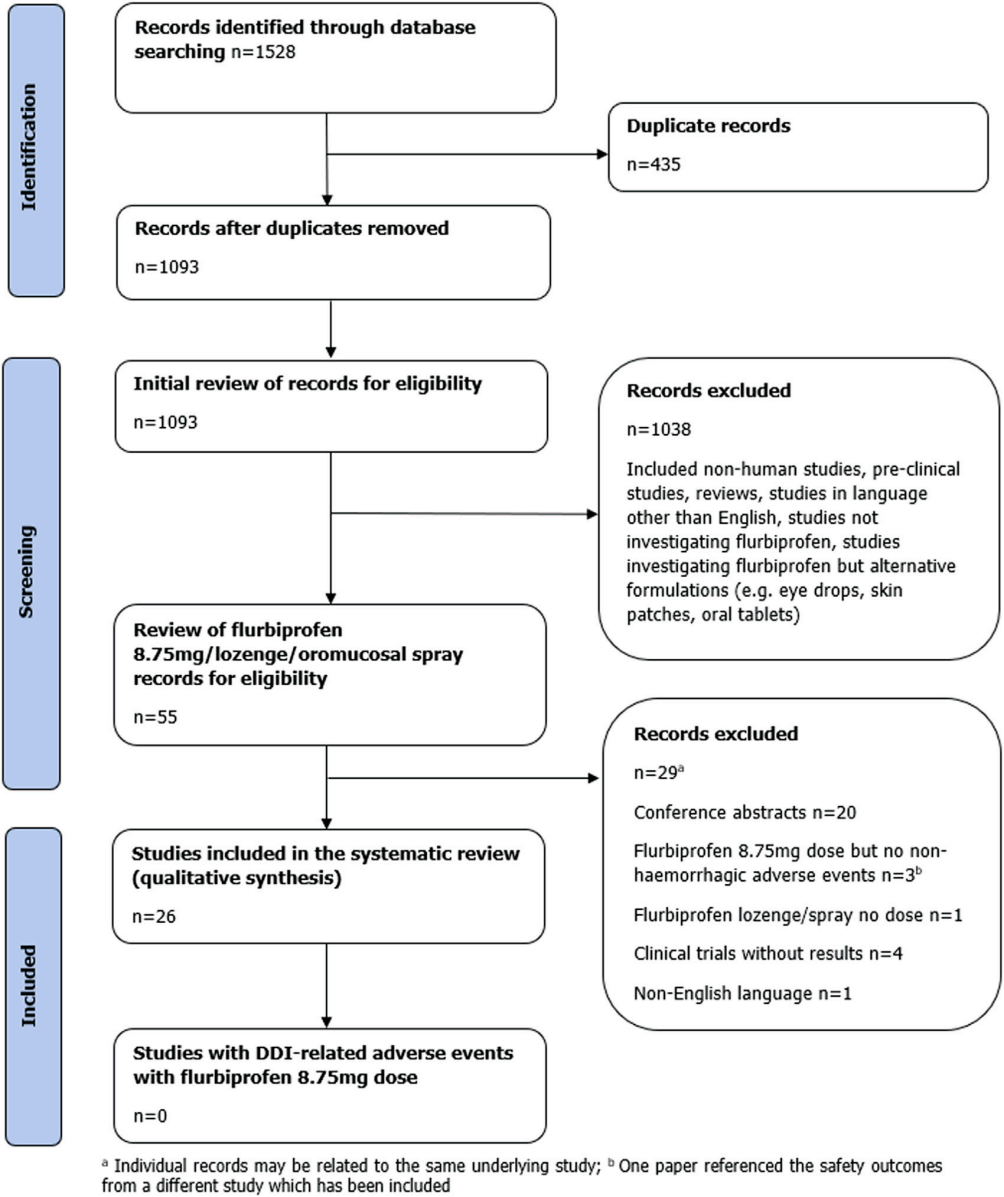
Flow diagram of study selection.

In total, 1,528 publications were identified by the search strategy across the individual electronic databases utilised (Figure 1). After de-duplication, 1,093 publications were screened for possible eligibility; 1,038 publications were deemed ineligible and were excluded at this stage (Figure 1). Further screening of the remaining 55 publications determined an additional 29 publications did not meet the eligibility criteria, and thus were excluded. Reason for exclusion have been listed in Figure 1.

Twenty-six publications met the inclusion criteria and were included in the final analysis. Of these, 23 studies were randomised controlled trials (RCTs), and three were case reports.

No additional studies were identified in the EMA database of case reports. Furthermore, it was not possible to identify relevant cases in EudraVigilance, due to lack of detail on formulation and/or dose of flurbiprofen. No additional relevant publications were identified through the manual search of reference lists. Four publications were identified using the initial search strategy which examined low doses of flurbiprofen (other than 8.75 mg); however these did not meet inclusion criteria and so were excluded (Heasman et al., 1989; Schachtel et al., 2002; Dionne et al., 2004; Cebi, 2020).

### Results of eligible studies

No adverse events resulting from DDIs were identified in the 26 studies included in this study, therefore the objectives could not be investigated further. The results of the 26 studies have been summarised to describe the reported non-haemorrhagic adverse events. Of the 26 studies, 14 studies detailed at least one non-haemorrhagic AE which the authors considered was possibly or probably treatment related (Table 2). It was not specified whether adverse events in the other 12 studies were related to flurbiprofen treatment (Supplementary Material: Table 1).

**TABLE 2:** Summary of studies reporting treatment-related non-haemorrhagic events with flurbiprofen 8.75 mg*.

Author, year/Trial number	Study design	Country	Study period	Population	Mean age (years)	Sex (% female)	Exposure	Comparator	Follow-up duration	Subjects (n)	Total AEs reported in flurbiprofen group	AEs reported
Benrimoj et al. (2001)	Randomised double-blind placebo-controlled trial	Australia	1996	Patients ≥18 years with sore throat due to URTI	Flurbiprofen 8.75 mg 19.8; 12.5 mg 20.2; Placebo 19.7	Flurbiprofen 8.75 mg 52.3%; 12.5 mg 51.6%; Placebo 57.8%	Flurbiprofen 8.75 mg lozenge	Similar taste placebo lozenge	5 days	Total n = 320 Flurbiprofen 8.75 mg n = 128; 12.5 mg n = 64; Placebo n = 128	96 from 66 patients (80 treatment-related)^a^	Most commonly reported treatment-related: Taste perversion, n = 34 (26.6%); headache, n = 17 (13.3%); dizziness, n = 5 (3.9%); paraesthesia, n = 6 (4.7%) and nausea, n = 2 (1.6%)
Blagden et al. (2002)	Randomised double-blind placebo-controlled trial	United Kingdom	1998	Patients ≥12 years with sore throat	Flurbiprofen 32.0; Placebo 32.4	Flurbiprofen 65.2%; Placebo 63.3%	Flurbiprofen 8.75 mg lozenge	Placebo lozenge	8 days	Total n = 459 Flurbiprofen n = 230; Placebo n = 229	95 treatment-related from 71 patients^a^	Most frequently reported treatment-related: Nausea, n = 11 (4.8%); dyspepsia, n = 2 (0.9%); diarrhoea, n = 10 (4.3%); dry mouth, n = 7 (3%); paraesthesia, n = 8 (3.5%); taste perversion, n = 24 (10.4%); and abdominal pain, n = 9 (3.9%)
Burova et al. (2018)	Randomised double-blind double-dummy active-controlled trial	Russia	2014–2015	Patients ≥18 years with moderate or severe sore throat due to URTI	Flurbiprofen 8.75 mg spray 41.6; 8.75 mg lozenge 42.1	Flurbiprofen 8.75 mg spray 60.8%; 8.75 mg lozenge 57.2%	Flurbiprofen 8.75 mg lozenge; 8.75 mg throat spray	Placebo lozenge; placebo throat spray	2 h	Total n = 439 Flurbiprofen 8.75 mg spray n = 217; 8.75 mg lozenge n = 222	96 TEAEs from Flurbiprofen spray group; 76 from Lozenge group^a^	NR (See Radkova)
Calapai et al. (2013)	Case report	Italy^b^	Not stated	N/A	29	0%	Flurbiprofen oral spray	N/A	N/A	n = 1	N/A	Fatal hypersensitivity reaction
de Looze et al. (2016)	Randomised double-blind placebo-controlled trial	Australia and New Zealand	2012	Patients ≥18 years with sore throat due to URTI	Flurbiprofen 25.5; Placebo 25.7	Flurbiprofen 41.8%; Placebo 46.1%	Flurbiprofen 8.75 mg flavoured spray	Placebo non-flavoured spray	1–4 days after Day 3	Total n = 505 Flurbiprofen n = 249; Placebo n = 256	19 TEAEs	Headache, n = 7, (2.8%); throat irritation, n = 6, (2.4%); abdominal discomfort, n = 1 (0.4%); dyspepsia, n = 1 (0.4%); chest discomfort, n = 1 (0.4%); ulcer, n = 1 (0.4%); seasonal allergy, n = 1 (0.4%); alcohol poisoning, n = 1 (0.4%)
di Meo et al. (2016)	Case report	Italy^b^	Not stated	N/A	40	100%	Flurbiprofen 8.75 mg oral	N/A	N/A	n = 1	N/A	Acute localized exanthematous pustulosis
Giovannini et al. (2017)	Case report	Italy	Not stated	N/A	10	100%	Flurbiprofen 8.75 mg tablet	N/A	N/A	n = 1	N/A	Anaphylaxis
Limb et al. (2009)	Open-label single dose three-way crossover pilot study	Not stated	Not stated	Healthy volunteers	Not stated	Not stated	Flurbiprofen 8.75 mg lozenge	0.3%, 0.15% Benzdyamine spray or gargle	45 min post-dose	Total n = 4	1 from 1 patient	Dry throat
Radkova et al. (2017)	Randomised double-blind double-dummy active controlled trial	Russia	2014–2015	Patients ≥18 years to ≤75 years with sore throat due to URTI	23	66.7%	Flurbiprofen 8.75 mg throat spray and placebo lozenge	Flurbiprofen 8.75 mg lozenge and placebo spray	24 h after dose (for primary outcome)	Safety set: Total n = 440 Flurbiprofen spray n = 218; lozenge n = 222 Per protocol (PP) set: Total n = 417 Flurbiprofen spray n = 205; lozenge n = 212	156 TEAEs from 96 patients in Flurbiprofen spray group; 130 from 79 patients in Lozenge group^a^	Treatment-related: Flurbiprofen 8.75 mg Spray group: Throat irritation, n = 5 (2.3%); dyspepsia, n = 3 (1.4%); malaise, n = 1 (0.5%); cough, n = 1 (0.5%); and hiccups, n = 1 (0.5%) Flurbiprofen 8.75 mg lozenge group: glossodynia, n = 2 (0.9%); tachycardia, n = 1 (0.5%); dyspepsia, n = 1 (0.5%); hypoesthesia, n = 1 (0.5%) and somnolence, n = 1 (0.5%)
Schachtel et al. (2014) NCT01048866	Randomised double-blind placebo-controlled trial	United States	2009–2011	Patients ≥18 years with sore throat	Flurbiprofen 33.5; Placebo 34.2	Flurbiprofen 60.4%; Placebo 59.8%	Flurbiprofen 8.75 mg lozenge	Placebo sugar based lozenge	24 h	Total n = 198 Flurbiprofen n = 101; Placebo n = 97	25.7% of patients in Flurbiprofen group (within 24 h of study start); 33.7% of exposed patients reported AEs within 7 days^a^	Related to URTI symptoms (headache and throat irritation); incidence of gastrointestinal treatment-related AEs was 3.0%
Schachtel et al. (2016) NCT01049334	Randomised double-blind placebo-controlled trial	United States	2011	Patients ≥18 years with acute pharyngitis	Flurbiprofen 19.8; Placebo 19.8	Flurbiprofen 52.9%; Placebo 61.8%	Flurbiprofen 8.75 mg lozenge	Placebo sugar based lozenge	7 days	Total n = 204 Flurbiprofen n = 102; Placebo n = 102	36 patients reported AEs. 20 patients reported a treatment-related AE.	Most common AEs in both the flurbiprofen 8.75 mg lozenge and placebo groups were stomatitis and oral pain, oral paresthesia, abdominal pain and nausea, tonsillitis and throat irritation, otitis media and headache; In both groups, patients reported the following gastrointestinal AEs including upper abdominal pain, oral pain, stomatitis, cheilitis, gingivitis and oral discomfort
Schachtel et al. (2018a) NCT01986361^c^	Randomised double-blind placebo-controlled trial	United States	2013–2014	Patients ≥18 years with sore throat	Flurbiprofen 19.5; Placebo 19.6	Flurbiprofen 57%; Placebo 62%	Flurbiprofen 8.75 mg lozenge	Placebo matched lozenge	3 h	Total n = 122 Flurbiprofen n = 101; Placebo n = 21	15 TEAEs from 10 patients	Abdominal discomfort* n = 1 (1%), diarrhoea n = 1 (1%), nausea n = 2 (2%), dizziness n = 1 (1%), headache n = 1 (1%), cough n = 1 (1%), throat irritation* n = 1 (1%), tonsillar hypertrophy n = 1 (1%), pyrexia n = 2 (2%), infections and infestations n = 2 (2%), conjunctivitis infective n = 1 (1%), and laryngitis n = 1 (1%)* considered possibly or probably related to treatment
Schachtel et al. (2018b) NCT01986361^c^	Randomised double-blind placebo-controlled trial	United States	2013–2014	Patients ≥18 years with sore throat due to URTI	Flurbiprofen 19.5; Placebo 19.6	Flurbiprofen 57%; Placebo 62%	Flurbiprofen 8.75 mg lozenge	Placebo matched lozenge	3 h	Total n = 122 Flurbiprofen n = 101; Placebo n = 21	TEAEs were reported by 10 patients^a^	Most common (affecting more than one patient in either treatment group): nausea n = 2 (2%); pyrexia n = 2 (2%); infections and infestations n = 2 (2%). Three AEs considered possibly or probably related to study treatment (abdominal discomfort and throat irritation)
Watson et al. (2000)	Randomised double-blind placebo-controlled trial	United Kingdom	1997	Patients ≥18 years with sore throat due to URTI	Total n = 301 Flurbiprofen 8.75 mg 27.7; 12.5 mg 28.4; Placebo 29.1	Flurbiprofen 8.75 mg 50.4%; 12.5 mg 55.8%; Placebo 51.2%	Flurbiprofen 8.75 mg lozenge	Placebo demulcent lozenge Flurbiprofen 12.5 mg lozenge	4 days	Total n = 301 Flurbiprofen 8.75 mg n = 129; 12.5 mg n = 43; Placebo n = 129	77 from 55 patients. 37 AE considered to be related to treatment^a^	Most commonly reported treatment-related AEs in the flurbiprofen 8.75 mg lozenge group were: taste perversion, n = 18 (13.9%); paraesthesia, n = 9 (7%); dry mouth, n = 1 (0.8%) and nausea, n = 2 (1.6%)

*Treatments and outcomes are reported as specified by authors in the individual studies.

^a^ Comprehensive details regarding the AEs were not provided for studies so these may include haemorrhagic as well as non-haemorrhagic events.

^b^ Not stated in paper but inferred from authors affiliation.

^c^ Publications for Schachtel et al. (2018a) and Schachtel et al. (2018b) were based on results from the same clinical trial. However, AE reporting differed in Schachtel et al. (2018a).

AE, adverse event; TEAE, Treatment-emergent adverse event; URTI, upper respiratory throat infection; NR, not reported; N/A = not applicable.

Overall, across the 26 studies, the adverse events stated to be treatment-related with flurbiprofen 8.75 mg were taste perversion, paraesthesia, headache, dizziness, nausea, dyspepsia, diarrhoea, dry mouth, dry throat, abdominal pain, abdominal discomfort, dry nipping throat, throat irritation, cough, hiccups, glossodynia, hypoesthesia, somnolence, malaise, tachycardia and gastrointestinal adverse events (reported in RCTs) and anaphylaxis, fatal hypersensitivity and acute localised exanthematous pustulosis (reported in case reports) (Table 2).

### Results of risk of bias assessment and certainty of evidence

Adverse events related to DDIs were not reported in any of the studies identified. Consequently, results relating to the primary objective of the systematic review were not available and thus these assessments for each individual study were not conducted.

## Discussion

The primary objective of this systematic review was to identify the frequency of all non-haemorrhagic events occurring as a result of concomitant use of flurbiprofen 8.75 mg dose with other medicinal products (i.e., DDIs). Secondary objectives included describing the nature of the drug interactions, the severity of the non-haemorrhagic event(s) and comparing frequencies of non-haemorrhagic events following exposure to flurbiprofen 8.75 mg in combination with other medicinal products, with the frequency of such events in a comparator group, where available. No adverse events resulting from DDIs were identified in the 26 studies included in this study, therefore the objectives could not be investigated further.

Amongst the 26 studies included in this systematic review, there were 21 distinct treatment-related adverse events reported (Table 2). With the exception of malaise and tachycardia, these treatment-related adverse events are in keeping with known adverse events listed in the SmPC for both flurbiprofen 8.75 mg lozenge and spray formulations (Reckitt Benckiser Healthcare UK Ltd, 2016; Reckitt Benckiser Healthcare UK Ltd, 2018). Tachycardia was the only cardiovascular treatment-related adverse event reported. Three case reports were included for consideration in the review, describing a fatal hypersensitivity reaction to oral flurbiprofen spray (Calapai et al., 2013), anaphylaxis to an oral 8.75 mg flurbiprofen tablet (Giovannini et al., 2017), and acute localised exanthematous pustulosis to oral 8.75 mg flurbiprofen (di Meo et al., 2016).

To identify additional adverse events resulting from possible DDIs when flurbiprofen was used concomitantly with another medication, a review of EudraVigilance data was conducted. However, it was not possible to determine dose and/or formulation of flurbiprofen in each reported event, therefore it was not possible to conduct further analyses using spontaneously reported data from this source. Further searches of EMA case reports were carried out, however no additional cases were identified.

Importantly, 23 of the 26 included studies were RCTs. RCTs are considered to be the gold standard for investigating efficacy of medicines, however there are recognised limitations for reporting adverse events in RCTs (Junqueira et al., 2021). In addition, external validity of the results of RCTs is often a limitation of this study design (Hariton and Locascio, 2018; He et al., 2020). One reason for this is the highly selected study population included in trials; strict exclusion criteria are usually applied, meaning non-healthy subjects are excluded. Most commonly applied exclusion criteria in clinical research relates to age, comorbidities, and concomitant medications (He et al., 2020). As most studies included in this systematic review were randomised controlled trials, this could be a reason that we did not identify any DDI-related adverse events in this analysis. Examination of the protocols for the randomised controlled trials included in this systematic review revealed that the exclusion criteria were broadly as per section 4.5 of the SmPC for flurbiprofen (Interaction with other medicinal products and other forms of interaction) i.e., NSAIDs, Anticoagulants, e.g., warfarin, lithium, mifepristone, quinolone antibiotics and alcohol (Reckitt Benckiser Healthcare UK Ltd, 2016; Reckitt Benckiser Healthcare UK Ltd, 2018; Reckitt Benckiser Healthcare (UK) Ltd, 2022). Real-world observational studies of flurbiprofen 8.75 mg in normal clinical use are required to determine the types, frequency, and severity of adverse events when flurbiprofen is used in combination with other medicinal products.

### Strengths

To date, although the increased risk of adverse events due to the interaction of flurbiprofen with other medicinal products are known, the authors are not aware of any published systematic review of the risk of non-haemorrhagic events occurring with flurbiprofen 8.75 mg lozenge/oromucosal spray as a result of DDIs. An extensive search was conducted across multiple databases using broad search terms for flurbiprofen including all synonyms for flurbiprofen and the formulation where this was possible. Both published and unpublished sources of data were reviewed with the aim of identifying all eligible studies, thereby reducing the likelihood of capturing a biased sample of studies. Eligibility was decided and data was extracted by two independent reviewers. A third reviewer adjudicated any discrepancies if these could not be resolved by discussion. Therefore, the potential for reporting bias was reduced.

A review of EudraVigilance and the EMA website was conducted in order capture any non-haemorrhagic adverse events which were not reported within the initial search of published studies. However, examination of EudraVigilance was restricted by the limited information available for dose and formulation of flurbiprofen.

### Limitations

This systematic review was part of a wider study with the aim to assess current evidence on adverse events (haemorrhagic and non-haemorrhagic) which occur with flurbiprofen 8.75 mg dose, in particular, as a result of interaction with other medicinal products. The search was conducted up to 28 April 2020 with the results focusing on haemorrhagic events published in 2021 (Dhanda et al., 2021). In order to include the most recent data for this publication, a literature review was conducted using the same search criteria to cover the period between 29 April 2020 to 14 February 2023. One newly published paper was identified (Schachtel et al., 2021). This publication only included data combined from two individual RCTs, both of which were already identified in the search conducted for this systematic review (Schachtel et al., 2014; Schachtel et al., 2016; Dhanda et al., 2021).

There are a number of limitations which have been described previously (Dhanda et al., 2021). Some limitations are inherent to the systematic review methodology, whereas others relate to limitations of the published studies which were eligible for inclusion.

Potential biases in the review process include only reviewing English language studies. The studies included were based in Australia, Europe, Russia, the United States, and New Zealand. It is possible that results may not be generalisable to other populations, however, it is not expected that flurbiprofen 8.75 mg would have a differing safety profile in other geographical regions. Conference abstracts were excluded from this review which may introduce publication bias. In addition, studies that are smaller and have few results, in terms of both efficacy and safety, may not be published. There are only a few published studies relating to the lower dose of flurbiprofen, i.e., 8.75 mg. Non-haemorrhagic events presented are those that were reported in publications, i.e., those that were available in the public domain. There is a potential for under-reporting and misclassification of DDI-related non-haemorrhagic adverse events in studies that were not specifically designed to investigate this outcome. The studies included in the systematic review were not specifically designed (and therefore not powered) to investigate non-haemorrhagic adverse events, in particular DDI-related non-haemorrhagic adverse events of flurbiprofen 8.75 mg dose with concomitant medication use. The findings from this systematic review may therefore not provide a true representation of the overall risk. It is possible that in open-label trials and observational studies, reporter and/or observer bias may have led to differential misclassification of the outcome whereby awareness that a low dose of flurbiprofen was being used may have led to a reporting bias, impacting patient and/or investigator data collection.

Finally, in terms of quality of evidence, risk of bias assessments were not performed as no DDI-related non-haemorrhagic events occurring as a result of concomitant use of flurbiprofen 8.75 mg dose (any formulation) with other medicinal products were identified.

## Conclusion

In conclusion, results from this first systematic review on the risk of non-haemorrhagic events occurring as a result of concomitant use of flurbiprofen 8.75 mg lozenge/oromucosal spray with other medical products did not provide evidence for DDI related adverse events.

Additional, appropriately designed research would be required to confirm whether these findings suggest a true absence of risk or limitations in reporting. Such studies could include observational post-authorisation safety studies (PASS) specifically designed to collect data on DDI-related non-haemorrhagic safety outcomes with flurbiprofen 8.75 mg dose. These ‘real-world’ studies may aid further investigation of any potential risk.

## Data Availability

The original contributions presented in the study are included in the article/Supplementary Material, further inquiries can be directed to the corresponding author.
