# Supplementary material for: A systematic review of flurbiprofen 8.75 mg dose and risk of adverse events (excluding haemorrhagic) resulting from drug-drug interactions
Source: Front Pharmacol. 2024 Mar 6;15:1107185. doi: 10.3389/fphar.2024.1107185 (PMC10952129; doi:10.3389/fphar.2024.1107185)
Supplement: Supplementary file 1 [file DataSheet1.PDF]

Supplementary Material

Inclusion criteria

Population

All patients (any age) taking flurbiprofen 8.75mg dose (any formulation) in any setting (primary and/or secondary care)
The population was not restricted to the UK and included international studies

Intervention (Exposure)

Reported use of flurbiprofen 8.75mg dose (any formulation) for any indication alone or in combination with other medicinal products

Comparator

A study comparator group was not required for inclusion. However, where a comparator group was specified, this could have been placebo or an active comparator. Active comparators could include NSAIDs:

- Of any formulation (i.e. oral, topical)
- Prescribed for any indication
- Within any of the three categories of sales (General sales list (GSL), Pharmacy only (PO), Prescription only medicines (POM)
- Prescribed alone
- Prescribed in combination with other medicinal products

The active comparator group could also include:

- Flurbiprofen 8.75mg dose (whichever is not the specific exposure formulation) alone
- Flurbiprofen 8.75mg dose (whichever is not the specific exposure formulation) plus other medicinal products
- Flurbiprofen 8.75mg dose (where it is the same as the specific exposure formulation) plus other medicinal products
- Flurbiprofen at any other dose (other than 8.75mg) alone and in any formulation
- Flurbiprofen at any other dose (other than 8.75mg) in any formulation plus other medicinal products

Outcomes

Studies were included if they reported any non-haemorrhagic adverse event. The non-haemorrhagic event could have occurred:

- In any anatomical site
- At any severity
- With flurbiprofen 8.75mg dose (any formulation) alone
- With flurbiprofen 8.75mg dose (any formulation) plus other medicinal products

• With the comparator drug alone (where comparator group available)<br>• With comparator drug plus other medicinal products (where comparator group available) Studies with non-haemorrhagic AEs reported only in the comparator arm (and not in the flurbiprofen arm) were considered for inclusion.
**Study Design**<br><br>The following study designs were eligible for inclusion in the systematic review<br>• Clinical trials (randomised and non-randomised, blinded and non-blinded)<br>• Cohort studies (prospective and retrospective),<br>• Case-control studies<br>• Cross sectional studies<br>• Case series<br>• Case reports

Search strategy

PubMed

Filters applied - English language, Humans

Search concept(s) -

(Flurbiprofen OR Strepfen OR Strepsils OR Strefen OR Benactiv Gola OR Strepflam OR

Graneodin-F OR СТРЕПФЕН OR Стрепсилс® Интенсив OR Strepsilsmaxpro OR Streflam OR

Verofen OR Buccostad OR Sorenex OR Geifen OR Camilfen OR Sereno OR Ultravox Maxe OR

Strefzap OR Benactivdol Gola OR Froben)

AND

(spray OR oral spray OR oromucosal spray OR mucosal spray OR mucosal OR oromucosal OR

lozenge* OR buccal OR sublingual OR pastille* OR topical OR granule* OR oral solution OR

mouthwash OR 8.75*)

Embase

Filters applied – English language, Humans, Articles

Search concept(s) -

(Flurbiprofen OR Strepfen OR Strepsils OR Strefen OR “Benactiv Gola” OR Strepflam OR

Graneodin-F OR СТРЕПФЕН OR “Стрепсилс® Интенсив” OR Strepsilsmaxpro OR Streflam

OR Verofen OR Buccostad OR Sorenex OR Geifen OR Camilfen OR Sereno OR “Ultravox Maxe”

OR Strefzap OR “Benactivdol Gola” OR Froben)

AND

(spray OR “oral spray” OR “oromucosal spray” OR “mucosal spray” OR mucosal OR oromucosal

OR lozenge* OR buccal OR sublingual OR pastille* OR topical OR granule* OR “oral solution”

OR mouthwash OR 8.75*)

<u>Web of Science</u>

Filters applied - English language, Articles

Search concept(s) -

(Flurbiprofen OR Strepfen OR Strepsils OR Strefen OR Benactiv Gola OR Strepflam OR

Graneodin-F OR СТРЕПФЕН OR Стрепсилс® Интенсив OR Strepsilsmaxpro OR Streflam OR

Verofen OR Buccostad OR Sorenex OR Geifen OR Camilfen OR Sereno OR Ultravox Maxe OR

Strefzap OR Benactivdol Gola OR Froben)

AND

(spray OR oral spray OR oromucosal spray OR mucosal spray OR mucosal OR oromucosal OR

lozenge* OR buccal OR sublingual OR pastille* OR topical OR granule* OR oral solution OR

mouthwash OR 8.75*)

<u>Cochrane</u>

Filters applied – Trials

Search concept(s) -

(Flurbiprofen OR Strepfen OR Strepsils OR Strefen OR Benactiv Gola OR Strepflam OR

Graneodin-F OR СТРЕПФЕН OR Стрепсилс® Интенсив OR Strepsilsmaxpro OR Streflam OR

Verofen OR Buccostad OR Sorenex OR Geifen OR Camilfen OR Sereno OR Ultravox Maxe OR

Strefzap OR Benactivdol Gola OR Froben)

AND

(spray OR oral spray OR oromucosal spray OR mucosal spray OR mucosal OR oromucosal OR

lozenge* OR buccal OR sublingual OR pastille* OR topical OR granule* OR oral solution OR

mouthwash OR 8.75*)

<u>Clinicaltrials.gov</u>

Filters applied- none

Search concept(s) -

Flurbiprofen

<u>EU clinical trials.gov</u>

Filters applied – none

Search concept(s) -

(Flurbiprofen OR Strepfen OR Strepsils OR Strefen OR Benactiv Gola OR Strepflam OR

Graneodin-F OR СТРЕПФЕН OR Стрепсилс® Интенсив OR Strepsilsmaxpro OR Streflam OR

Verofen OR Buccostad OR Sorenex OR Geifen OR Camilfen OR Sereno OR Ultravox Maxe OR

Strefzap OR Benactivdol Gola OR Froben)

AND

(spray OR oral spray OR oromucosal spray OR mucosal spray OR mucosal OR oromucosal OR

lozenge* OR buccal OR sublingual OR pastille* OR topical OR granule* OR oral solution OR

mouthwash OR 8.75*)

<u>PubMed case reports</u>

Filter applied- English language, Humans, Case reports

Search concept(s) -

(Flurbiprofen OR Strepfen OR Strepsils OR Strefen OR Benactiv Gola OR Strepflam OR

Graneodin-F OR СТРЕПФЕН OR Стрепсилс® Интенсив OR Strepsilsmaxpro OR Streflam OR

Verofen OR Buccostad OR Sorenex OR Geifen OR Camilfen OR Sereno OR Ultravox Maxe OR

Strefzap OR Benactivdol Gola OR Froben)

Table 1: Summary of studies reporting non-haemorrhagic events (not treatment-related) with flurbiprofen 8.75mg*

Author / Trial Number	Study Design	Country	Study Period	Population	Age (mean) years	Sex[a] (%)	Exposure	Comparator	Follow-up duration	Subjects (n)	Number patients reporting an AE in Flurbiprofen Group	AEs reported
(ClinicalTrials.gov, 2009b) NCT01048866	Randomised double-blind placebo-controlled trial	USA	2011	Patients ≥ 18 years with sore throat due to acute pharyngitis	Flurbiprofen 33.5 years Placebo 34.2 years	Flurbiprofen 60.4% f, 39.6% m Placebo 59.8% f, 40.2% m	Sugar-based, flavoured flurbiprofen 8.75mg lozenge	Sugar-based, flavoured matching placebo lozenge	7 days	Total n=198 Flurbiprofen n=101 Placebo n=97	34[b]	Abdominal pain upper 1/101 (0.99%), Aphthous stomatitis 1/101 (0.99%), Diarrhoea 2/101 (1.98%), Dyspepsia 1/101 (0.99%), Flatulence 1/101 (0.99%), Gingival erythema 1/101 (0.99%), Mouth ulceration 1/101 (0.99%), Nausea 1/101 (0.99%), Oropharyngeal blistering 1/101 (0.99%), Paraesthesia oral 1/101 (0.99%), Vomiting 1/101 (0.99%), Chills 1/101 (0.99%), Pain 1/101 (0.99%), Pyrexia 2/101 (1.98%), Conjunctivitis viral 1/101 (0.99%), Influenza 1/101 (0.99%), Oral

												herpes 1/101 (0.99%), Otitis media 1/101 (0.99%), Pharyngitis streptococcal 1/101 (0.99%), Flank pain 1/101 (0.99%), Musculoskeletal pain 1/101 (0.99%), Dizziness 2/101 (1.98%), Headache 8/101 (7.92%), Paraesthesia 4/101 (3.96%), Cough 4/101 (3.96%), Dry throat 1/101 (0.99%), Nasal congestion 1/101 (0.99%), Pharyngeal hypoaesthesia 1/101 (0.99%), Productive cough 1/101 (0.99%), Throat irritation 10/101 (9.90%).
(ClinicalTrials.gov, 2009a) NCT01049334	Randomised double-blind placebo-controlled trial	USA	2011	Patients ≥ 18 years with painful pharyngitis	Flurbiprofen 19.8 years Placebo 19.8 years	Flurbiprofen 52.9% f, 47.1% m Placebo 61.8% f, 38.2% m	Flurbiprofen 8.75mg lozenge	Placebo lozenge	7 days	Total n=204 Flurbiprofen n=102 Placebo n=102	34	Tinnitus 1/102 (0.98%), Conjunctivitis 1/102 (0.98%), Ocular hyperaemia 1/102 (0.98%), Abdominal pain upper 3/102 (2.94%), Aphthous stomatitis 3/102

												(2.94%), Oral pain 3/102 (2.94%), Nausea 2/102 (1.96%), Stomatitis 2/102 (1.96%), Cheilitis 1/102 (0.98%), Gingivitis 1/102 (0.98%), Oral discomfort 1/102 (0.98%), Fatigue 1/102 (0.98%), Tonsillitis 4/102 (3.92%), Otitis media 3/102 (2.94%), Bronchitis 1/102 (0.98%), Bite 1/102 (0.98%), Joint stiffness 1/102 (0.98%), Muscle twitching 1/102 (0.98%), Paraesthesia 4/102 (3.92%), Headache 3/102 (2.94%), Paraesthesia oral 2/102 (1.96%), Somnolence 1/102 (0.98%), Throat irritation 3/102 (2.94%), Cough 2/102 (1.96%), Nasal congestion 1/102 (0.98%), Nasal dryness 1/102 (0.98%), Oropharyngeal swelling 1/102 (0.98%), Rhinorrhoea

												1/102 (0.98%), Sneezing 1/102 (0.98%), Cold sweat 1/102 (0.98%) and Urticaria 1/102 (0.98%).
(ClinicalTrials.gov, 2013) NCT01986361	Randomised, double-blind, placebo-controlled study	USA	2014	Patients ≥ 18 years with acute pharyngitis or sore throat	Flurbiprofen 19.5 years Placebo 19.6 years	Flurbiprofen 57.4% f, 42.6% m Placebo 61.9% f, 38.1% m	Flurbiprofen 8.75mg lozenge	Placebo vehicle lozenge	3 hours	Total n=122 Flurbiprofen n=101 Placebo n=21	10	Abdominal discomfort 1/101 (0.99%), Diarrhoea 1/101 (0.99%), Nausea 2/101 (1.98%), Pyrexia 2/101 (1.98%), Conjunctivitis infective 1/101 (0.99%), Laryngitis 1/101 (0.99%), Dizziness 1/101 (0.99%), Headache 1/101 (0.99%), Cough 1/101 (0.99%), Throat irritation 1/101 (0.99%) and Tonsillar hypertrophy 1/101 (0.99%).
(Euctr, 2010) 2010-022899-32	Open-label, randomised, 5-way crossover study	UK	2011	Patients > 18 years to <55 years	25.4 years	50% f, 50% m	Flurbiprofen spray (15ml) 4 different flavours (Treatment B, C, D, E)	Flurbiprofen 8.75mg lozenge (Treatment A)	5 weeks	Total n=12	8	Treatment A: Dizziness postural, n=1 (8%); dry skin, n=1 (8%). Treatment B: Upper respiratory tract infection, n=3 (25%); arthralgia, n=1 (8%) Treatment C: Headache, n=1

												(8%); rash macular, n=1 (8%)<br>Treatment D: Chapped lips, n=1 (8%); cheilitis, n=1 (8%); nausea, n=1 (8%); dizziness postural, n=1 (8%); rash, n=1 (8%)<br>Treatment E: Cheilitis, n=1 (8%); lip dry, n=1 (8%); rash pustular, n=1 (8%); headache, n=1 (8%); rash maculo-papular, n=1 (8%)
(Euctr, 2007) 2006-006769-17	Randomised, controlled, open-label study	UK	2008	Patients ≥ 16 years to <65 years with sore throat	Flurbiprofen 33.5 years Ibuprofen 34.8 years	Flurbiprofen 45.5% f, 54.5% m Ibuprofen 75.0% f, 25.0% m	Flurbiprofen 8.75mg lozenge	Ibuprofen oral caplets 200mg (2 caplets)	12 hours	Total n=23 Flurbiprofen n=11 Ibuprofen n=12	N/A	No non-haemorrhagic AEs reported in the flurbiprofen 8.75mg lozenge treatment arm.
(Euctr, 2012) 2011-005848-10	Multicentre, randomised study	France, Germany, UK	2012	Patients ≥ 18 years with sore throat or tonsillopharyngitis	Overall, 32.8 years	Overall 68.8% f, 31.2% m	Ibuprofen lozenges (15mg, 25mg, 35mg)	Flurbiprofen 8.75mg lozenge or Placebo lozenge	2 days	Total n=186 Ibuprofen lozenges 15mg n=37, 25mg n=37, 35mg n=39 Flurbiprofen n=36 Placebo n=37	N/A	No non-haemorrhagic AEs reported in the flurbiprofen 8.75 mg lozenge (Strefen®) treatment arm.
(Aspley et al., 2016) NCT01048866	Randomised double-blind placebo-controlled trial	USA	2009-2011	Patients ≥ 18 years with sore throat described as swollen and inflamed	Flurbiprofen 19.8 years, Placebo 19.6 years	Flurbiprofen 42.4% f, 57.6% m	Flurbiprofen 8.75mg lozenge	Placebo lozenge	24 hours	Total n=124 Flurbiprofen n=59 Placebo n=65	15b	Most common: headache, n=1 (1.7%); nausea, n=2 (3.4%); upper abdominal

						Placebo 64.6 % f, 35.4% m						pain, n=3 (5.1%); paresthesia, n=2 (3.4%); paresthesia oral, n=2 (3.4%).
(Matzneller et al., 2012)	Randomised two-period cross-over open label trial	Austria	2010	Healthy volunteers aged 18-55 years	Per Protocol set: Flurbiprofen spray 41.4 years, lozenge 41.7 years	Per Protocol set: Flurbiprofen spray 60.5% f, 39.5% m, lozenge 57.5% f, 42.5% m	Flurbiprofen 8.75mg compressed lozenges	Flurbiprofen 8.75mg lozenges	3 days	Total n=12	4[b]	Rhinopharyngitis, n=2 (16.7%), gastroenteritis, n=1 (8.3%); herpes simplex infection, n=1 (8.3%); clinically relevant erythrocyte sedimentation rate increase, n=1 (8.3%); phlebitis, n=1 (8.3%)
(Russo et al., 2013)	Randomised double-blind placebo-controlled trial	Australia	2009	Patients ≥ 18 years to ≤75 years with sore throat due to URTI a	Flurbiprofen 30.8 years Placebo 30.4 years	Not stated	Flurbiprofen 8.75mg microgranules (sachet)	Matching placebo	3 days	Total n=373 Flurbiprofen n=186 Placebo n=187	23.1%[b]	Most common: headache, n=26 (14.0%)
(Schachtel et al., 2014) NCT01049334	Randomised double-blind placebo-controlled trial	USA	2011	Patients ≥ 18 years with acute sore throat and odynophagia	Flurbiprofen 19.8 years Placebo 19.8 years	Flurbiprofen 52.9% f, 47.1% m Placebo 61.8% f, 38.2% m	Flurbiprofen 8.75mg lozenge	Placebo sugar based lozenge	24 hours	Total n = 204 Flurbiprofen n=102 Placebo n=102	15[b]	Most common: nausea (n=1, 1%); headache (n=1, 1%)
(Shephard et al., 2015) NCT01049334 and NCT01048866	Randomised double-blind placebo-controlled trial	USA	2009-2011	Patients ≥ 18 years with acute sore throat and diagnosis of pharyngitis	Flurbiprofen 26.6 years Placebo 26.8 years	Flurbiprofen 56.7% f, 43.3% m Placebo 60.8% f, 39.2% m	Flurbiprofen 8.75mg lozenge	Placebo sugar based lozenge	7 days	Total n=402 Flurbiprofen n=203 Placebo n=199	24.1%[b]	Most common: headache (3.0%), throat irritation (5.9%), nausea (1.5%), paraesthesia (3.9%) and gastrointestinal complaints (7.9%). It is not reported whether these events were related to treatment with

												flurbiprofen 8.75 mg lozenge.
(Unknown, 2007)	Unknown	France	Not stated	Unknown	Not stated	Not stated	Flurbiprofen 8.75mg lozenge	Unknown	Not stated	Total N=459	NR	'The main trial, involving 459 patients …… the most frequent adverse effect was altered taste'

[*] Treatments and outcomes are reported as specified by authors in the individual studies

[a] f=female, m=male

[b] Comprehensive details regarding the AEs were not provided so these may include haemorrhagic as well as non-haemorrhagic events.

AE=adverse event, TEAE=Treatment-emergent adverse event, NR=not reported

REFERENCES

ASPLEY, S., SHEPHARD, A., SCHACHTEL, E., SANNER, K., SAVINO, L. & SCHACHTEL, B. 2016. Efficacy of flurbiprofen 8.75 mg lozenge in patients with a swollen and inflamed sore throat. *Curr Med Res Opin,* 32**,** 1529-38.

CLINICALTRIALS.GOV. 2009a. *A Randomized, Double-Blind, Placebo-Controlled Multiple-Dose Study to Determine the Efficacy, Onset, and Duration of Action of Flurbiprofen 8.75 mg Lozenge Compared to Its Vehicle Control Lozenge in Patients With Painful Pharyngitis (NCT01049334). 2009 [cited April 2020].* [Online]. Available: https://clinicaltrials.gov/ct2/show/NCT01049334 [Accessed].

CLINICALTRIALS.GOV 2009b. A Repeat-Dose, Multi-Centre, Randomized, Double-Blind, Placebo-Controlled, Study to Determine the Safety and Efficacy of Flurbiprofen 8.75 mg Lozenge Compared to Its Vehicle Control Lozenge in Patients With Painful Pharyngitis (NCT01048866). *Clinicaltrials.gov [17 july 2013]*.

CLINICALTRIALS.GOV 2013. A Single-Dose, Randomized, Double-Blind, Placebo-Controlled Onset-of-Action Study Utilizing the Double-Stopwatch Method (NCT01986361). *https://clinicaltrials.gov/show/NCT01986361*.

EUCTR, G. B. 2007. A randomised, controlled, open-label, single-centre, parallel group, pilot study to investigate the onset of action of a lozenge compared with a caplet in patients with sore throat. - Strepsils onset of action study. *http://www.who.int/trialsearch/Trial2.aspx?TrialID=EUCTR2006-006769-17-GB*.

EUCTR, G. B. 2010. An open label, randomised, single dose, 5 way cross over study to compare the rate and extent of absorption of a 8.75mg Flurbiprofen lozenge with flavour and excipient base variants of a 8.75mg Flurbiprofen spray. - Flurbiprofen 5 way Pilot PK Study. *http://www.who.int/trialsearch/Trial2.aspx?TrialID=EUCTR2010-022899-32-GB*.

EUCTR, G. B. 2012. Analgesic profile of 3 new Ibuprofen lozenges (V0498TA01A 15mg, 25mg, 35mg) after single administration in acute sore throat pain. *http://www.who.int/trialsearch/Trial2.aspx?TrialID=EUCTR2011-005848-10-GB*.

MATZNELLER, P., BURIAN, A., MARTIN, W., ANNONI, O., LAURO, V., TACCHI, R., BRUNNER, M. & ZEITLINGER, M. 2012. A randomised, two-period, cross-over, open-label study to evaluate the pharmacokinetic profiles of single doses of two different flurbiprofen 8.75-mg lozenges in healthy volunteers. *Pharmacology,* 89**,** 188-91.

RUSSO, M., BLOCH, M., DE LOOZE, F., MORRIS, C. & SHEPHARD, A. 2013. Flurbiprofen microgranules for relief of sore throat: a randomised, double-blind trial. *Br J Gen Pract,* 63**,** e149-55.

SCHACHTEL, B., ASPLEY, S., SHEPHARD, A., SHEA, T., SMITH, G., SANNER, K., SAVINO, L., REZUKE, J. & SCHACHTEL, E. 2014. Onset of action of a lozenge containing flurbiprofen 8.75 mg: a randomized, double-blind, placebo-controlled trial with a new method for measuring onset of analgesic activity. *Pain,* 155**,** 422-8.

SHEPHARD, A., SMITH, G., ASPLEY, S. & SCHACHTEL, B. P. 2015. Randomised, double-blind, placebo-controlled studies on flurbiprofen 8.75 mg lozenges in patients with/without group A or C streptococcal throat infection, with an assessment of clinicians' prediction of 'strep throat'. *Int J Clin Pract,* 69**,** 59-71.

UNKNOWN 2007. Flurbiprofen: new indication. Lozenges: NSAIDs are not to be taken like sweets! *Prescrire Int,* 16**,** 13.
